# Maintaining network activity in submerged hippocampal slices: importance of oxygen supply

**DOI:** 10.1111/j.1460-9568.2008.06577.x

**Published:** 2009-01

**Authors:** Norbert Hájos, Tommas J Ellender, Rita Zemankovics, Edward O Mann, Richard Exley, Stephanie J Cragg, Tamás F Freund, Ole Paulsen

**Affiliations:** 1 Department of Cellular and Network Neurobiology, Institute of Experimental Medicine, Hungarian Academy of SciencesSzigony u. 43, 1083 Budapest, Hungary; 2 Department of Physiology, Anatomy and Genetics, University of OxfordOxford, UK

**Keywords:** GABAergic interneuron, gamma oscillation, hippocampus, *in vitro*, rodent, sharp wave–ripple oscillation

## Abstract

Studies in brain slices have provided a wealth of data on the basic features of neurons and synapses. In the intact brain, these properties may be strongly influenced by ongoing network activity. Although physiologically realistic patterns of network activity have been successfully induced in brain slices maintained in interface-type recording chambers, they have been harder to obtain in submerged-type chambers, which offer significant experimental advantages, including fast exchange of pharmacological agents, visually guided patch-clamp recordings, and imaging techniques. Here, we investigated conditions for the emergence of network oscillations in submerged slices prepared from the hippocampus of rats and mice. We found that the local oxygen level is critical for generation and propagation of both spontaneously occurring sharp wave–ripple oscillations and cholinergically induced fast oscillations. We suggest three ways to improve the oxygen supply to slices under submerged conditions: (i) optimizing chamber design for laminar flow of superfusion fluid; (ii) increasing the flow rate of superfusion fluid; and (iii) superfusing both surfaces of the slice. These improvements to the recording conditions enable detailed studies of neurons under more realistic conditions of network activity, which are essential for a better understanding of neuronal network operation.

## Introduction

Much insight into the cellular basis of brain function stems from experiments conducted in acute brain slices *in vitro* (Yamamoto & McIlwain, 1966; Skrede & Westgaard, 1971). However, there are notable differences between brain slices and the intact brain in both the amount and patterns of activity, especially in relation to the rhythmic synchronous neuronal events as reflected in the electroencephalogram (Steriade, 2001). Studies of neurons and synapses under more realistic conditions would benefit from *in vitro* preparations retaining local network activity. Recently, by altering the ionic composition of the superfusion media, or by the addition of pharmacological agents, attempts have been made to capture naturalistic network activity in brain slice preparations. This has been particularly successful in interface-type slice chambers, in which brain slices are held at the interface between artificial cerebrospinal fluid (ACSF) and humidified gas (e.g. Fisahn *et al.*, 1998; Sanchez-Vives & McCormick, 2000; Kubota *et al.*, 2003; Maier *et al.*, 2003). Maintaining physiologically relevant network activity in submerged slices has proven much harder, but, if successful, would offer important experimental advantages over interface conditions, including faster exchange of pharmacological agents, visually guided patch-clamp recordings and advanced imaging techniques. Previously, synchronous network activity in submerged slices has been recorded only transiently (McMahon *et al.*, 1998; Kawaguchi, 2001; Gloveli *et al.*, 2005). Recently, however, sustained network oscillations were successfully recorded in submerged hippocampal slices at increased flow rates of superfusion solution (Hájos *et al.*, 2004; Mann *et al.*, 2005; Wu *et al.*, 2005).

Here, we explored conditions conducive to the emergence of sharp wave*–*ripple oscillations and fast oscillations in submerged hippocampal slices. We found that the local oxygen level in the superfusion fluid is a critical factor for the generation and propagation of network activity. In addition, we describe ways to improve oxygen supply to submerged brain slices, including the use of a new type of slice chamber with dual-surface superfusion.

## Materials and methods

**Fig. 1:**
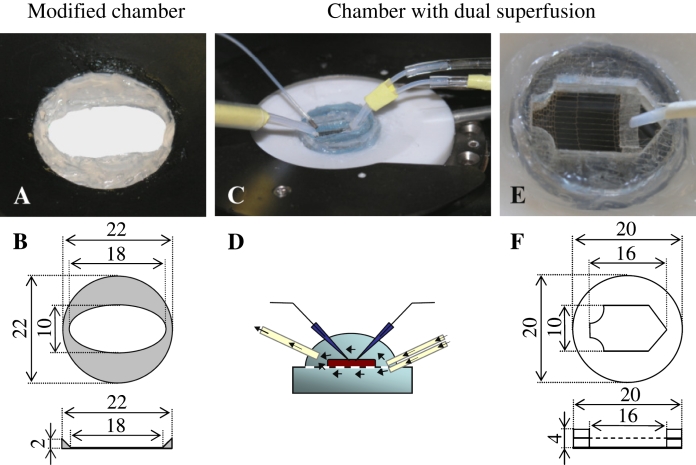
Modified submerged slice chambers with single and dual superfusion. (A) Commercially available standard submerged slice chamber modified with an inert plastic insert to optimize the flow of artificial cerebrospinal fluid (ACSF) across the slice. (B) Scaled drawings of the top view and the cross-section of the chamber insert (in mm). (C) Low magnification of a submerged slice chamber with two fluid inlets and one outlet. (D) Schematic diagram of the flow in the dual superfusion chamber. (E) Picture taken at higher magnification of a chamber insert developed for dual superfusion. In this design, the slices were placed on a mesh glued between two plastic rings with a thickness of 2 mm. Two separate fluid inlets allowed ACSF to flow separately above and below the slice. (F) Scaled drawing (in mm) of the insert shown in E.

All experiments were carried out in accordance with the UK Animals (Scientific Procedures) Act (1986) and the Hungarian Act of Animal Care and Experimentation (1998, XXVIII, section 243/1998), and with the guidelines of the institutional ethical code. Male Wistar rats (postnatal day 14–20; Harlan UK, Bicester, UK, or Charles River Hungary, Budapest) or CD1 mice (postnatal day 16–18; Charles River, Hungary, Budapest) were deeply anaesthetized with isoflurane and decapitated. Following decapitation, the brain was quickly removed into ice-cold cutting solution. Transverse hippocampal slices 400–450 μm in thickness were prepared using a Leica VT1000S microtome (Leica, Nussloch, Germany), and kept in an interface-type holding chamber at room temperature for at least 60 min before recording in standard or modified ACSF. The standard ACSF was composed of 126 mm NaCl, 2.5 mm KCl, 1.25 mm NaH_2_PO_4_, 2 mm MgCl_2_, 2 mm CaCl_2_, 26 mm NaHCO_3_, and 10 mm glucose, prepared with ultrapure water and bubbled with 95% O_2_/5% CO_2_ (carbogen gas), pH 7.2–7.4. All experiments were performed using rat hippocampal slices, except the investigation of the propagation of network activities from CA3 to CA1, which was performed in slices prepared from mice. Recordings were made in either an ‘Oslo’-style interface chamber or in commercially available submerged-type slice chambers (Luigs & Neumann, Ratingen, Germany, and MED64 probes, Alpha MED Sciences, Osaka, Japan). In preliminary experiments, we found that persistent oscillations in these conventional submerged-type slice chambers were only achieved with a flow rate exceeding 10 mL/min, similar to previous observations of hippocampal network activity (Wu *et al.*, 2005). Adding a dye to the superfusion fluid to visualize the flow, we noticed that the solution tended to flow along the edges of these chambers. The chamber design was therefore modified in either of two ways. First, in order to reduce the volume of the chamber and direct the superfusion fluid over the slice, an inert plastic insert was used (Fig. 1A and B). These plastic inserts were used in all experiments in which the effect of flow rate on generation of network oscillations was investigated. The second modification allowed a double superfusion system to be used (Supertech Ltd, Pecs, Hungary; http://www.super-tech.eu). In this design, the slices were placed on a mesh glued between two plastic rings with a thickness of 2 mm. Two separate fluid inlets allowed ACSF to flow separately above and below the slice (Fig. 1C–F). This second design was only used to study the propagation of network activity from CA3 to CA1.

### Measurement of oxygen saturation in the superfusate

The local oxygen saturation of the ACSF was measured 50–100 μm vertically above the CA3 region of the slice with an optode (tip diameter ∼50 μm; Microx TX3, PreSens GmbH, Germany). Vertical adjustment of the optode between 50 and 100 μm above the slice did not cause any substantial change in measured values at a given flow rate. The sensor was calibrated as follows: 2–3 mm sodium sulfite (Na_2_SO_3_) was used to eliminate dissolved oxygen from non-bubbled ACSF, to give 0% oxygen, and 95% oxygen was achieved in ACSF by bubbling for 1 h with 95% O_2_/5% CO_2_. Thus, the maximal *P*o_2_ in ACSF at room temperature was estimated to be ∼720 Torr. In a subset of experiments, ACSF bubbled with a mixture of 95% N_2_/5% CO_2_ was used to change the oxygen level in the chamber while retaining a constant flow rate.

### Measurement of oxygen saturation within the slice tissue

Oxygen was measured within submerged hippocampal slices using fast-scan cyclic voltammetry at carbon-fibre microelectrodes (fibre diameter 7 μm, tip length 20–30 μm, fabricated in-house) and a Millar voltammeter (J. Millar, Barts & the London School of Medicine and Dentistry, UK), using methods similar to those described previously for the detection of other electroactive biological substances, e.g. dopamine (Cragg & Greenfield, 1997; Cragg, 2003; Exley *et al.*, 2008). For the detection of oxygen specifically, the applied voltage was a triphasic waveform scanning from 0.0 to +0.8 V to −1.4 V and back to 0.0 V (vs. Ag/AgCl), as described previously (Venton *et al.*, 2003), with a scan rate of 880 V/s and a sampling frequency of 8 Hz. The peak reduction current for oxygen was detected between −1.3 and −1.4 V. The bath temperature was 32 °C. Currents due to oxygen were determined after subtraction of the background current that results from the charging of the electrode as well as exposure to the brain tissue environment. Currents were normalized to the current observed in solution at a flow rate of 6 mL/min, which was subsequently set to 80% saturation as measured with the optode at this perfusion speed.

Measurements were made outside of the tissue (50–100 μm above the surface) as well as 50 and 150 μm below the upper surface of the slice in the CA3 pyramidal cell layer. Perfusion speeds were 6, 3 or 1.8 mL/min. For a given flow rate and electrode depth, a steady-state background measurement was first obtained in oxygen-free ACSF (bubbled with 95% N_2_/5% CO_2_); oxygen-saturated buffer (bubbled with 95% O_2_/5% CO_2_) was then applied, and the increase in detected oxygen was recorded. The slice was thoroughly superfused with oxygen-free ACSF between each change in flow rate or change in electrode placement.

### Sharp wave–ripple oscillations

Slicing of rat hippocampus was performed in cutting solution containing 124 mm NaCl, 3 mm KCl, 1.25 mm KH_2_PO_4_, 5 mm MgSO_4_, 3.4 mm CaCl_2_, 26 mm NaHCO_3_, and 10 mm glucose, pH 7.2–7.4, bubbled with carbogen gas. Storage and recording were done in modified ACSF containing 124 mm NaCl, 3 mm KCl, 1.25 mm KH_2_PO_4_, 1 mm MgSO_4_, 3 mm CaCl_2_, 26 mm NaHCO_3_, and 10 mm glucose. Slices were mounted on planar 8 × 8 microelectrode arrays (electrode size, 50 × 50 μm; interpolar distance, 150 μm; Panasonic MED-P2105, Alpha MED Sciences), and maintained in a submerged condition at 32 °C, superfused with modified ACSF at 1.2–6 mL/min. Spontaneous field potentials from all 64 recording electrodes were acquired at 5 kHz, using the Panasonic MED64 system (Alpha MED Sciences).

Data were analysed off-line using IGOR Pro (Wavemetrics, Lake Oswego, OR, USA). Continuous MED64 recordings were divided into 10-s segments. Each segment was analysed for the incidence and amplitude of sharp waves. For sharp wave detection, recordings were filtered between 0.1 and 20 Hz, and sharp waves were detected as voltage fluctuations of more than two standard deviations above the baseline value, with a duration of more than 40 ms.

### Cholinergically induced oscillations and unit recordings

Cutting solution for preparing slices from rat or mouse hippocampus contained 252 mm sucrose, 2.5 mm KCl, 26 mm NaHCO_3_, 1 mm CaCl_2_, 5 mm MgCl_2_, 1.25 mm NaH_2_PO_4_, and 10 mm glucose, bubbled with carbogen gas. Standard ACSF was used for storage and recording. Oscillations were induced by bath application of 20 μm carbachol, and acquired at room temperature unless otherwise stated, using the MED64 planar multielectrode array system or a patch pipette containing ACSF. Spiking activity of individual cells was monitored extracellularly using a patch pipette filled with ACSF (Hájos *et al.*, 2004). Power spectra were estimated from 10-s traces with the Welch method using 1-s-wide time windows with 50% overlap. Oscillatory power between 10 and 20 Hz for measurements at room temperature was calculated with a Morlet wavelet. Spike rates were binned into 1-s time windows and averaged.

### Statistical analyses

Statistical analyses were performed using Origin 7.5 software (OriginLab Corporation, Northampton, MA, USA). Data are presented as mean ± SD, unless otherwise indicated. Datasets were compared by independent or paired Student’s *t*-test, as appropriate in each case. Pearson’s product–moment correlation coefficient was used to estimate correlations between variables.

## Results

**Fig. 2:**
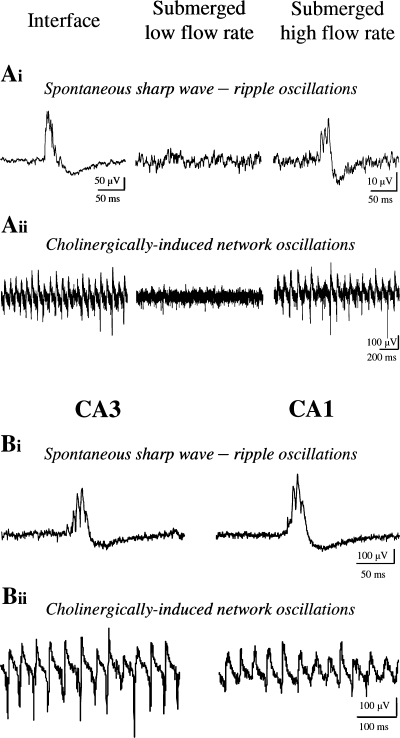
Generation and propagation of network events in submerged slice chambers. (A) Comparison of network activity recorded from hippocampal slices in an ‘Oslo’-style interface chamber and a modified submerged slice chamber with standard superfusion at low and high flow rates. (i) Spontaneous network activity recorded in an interface chamber (left), a submerged slice chamber at a low flow rate of 1.9 mL/min (middle), and a high flow rate of 5.2 mL/min (right). Note sharp wave*–*ripple activity in the interface chamber and only at a high flow rate in the submerged slice chamber. (ii) Cholinergically induced network activity in an interface chamber (left), and in a submerged slice chamber at a low flow rate (middle) and at a high flow rate (right). Note network oscillations in the interface chamber and only at a high flow rate in the submerged slice chamber. These recordings were made extracellularly in the pyramidal cell layer of CA3 of transverse hippocampal slices prepared from postnatal day 14–20 Wistar rats. Spontaneous sharp wave*–*ripple activity was recorded in slightly modified ACSF (see Materials and methods). After induction of fast network oscillations by bath application of 20 μm carbachol in standard ACSF, recordings were taken after 15 min. Sharp wave*–*ripple events were digitally bandpass filtered between 0.1 and 500 Hz; fast network oscillations were low-pass filtered at 2 or 5 kHz. (B) Propagation of network events in a modified submerged slice chamber with dual superfusion. Sample traces of sharp wave*–*ripples (i) and cholinergically induced fast network oscillations (ii) recorded simultaneously in CA3 and CA1 of mouse hippocampal slices. These recordings were made in standard ACSF with a flow rate of 3–3.5 mL/min for each channel at 30–32 °C. The incidence and the peak amplitude of spontaneous sharp wave*–*ripples were comparable in both hippocampal regions (CA3, 1.3 ± 0.8 Hz and 418 ± 100 μV; CA1, 1.2 ± 0.9 Hz and 350 ± 118 μV; *n*=6; *P*>0.1, independent Student’s *t*-test). In the case of fast oscillations, the frequency of the network activity was not different (CA3, 30.4 ± 2.2 Hz; CA1, 30.8 ± 2.1 Hz; *n*=6; *P*>0.1, independent Student’s *t*-test), whereas the mean peak power was significantly smaller in CA1 than in CA3 (CA3, 275 ± 120 μV^2^/Hz; CA1, 41 ± 17 μV^2^/Hz; *n*=6; *P*<0.05, independent Student’s *t*-test).

To compare network activity in submerged and interface conditions, we started by recording spontaneous sharp wave*–*ripples and cholinergically induced network oscillations in the CA3 region of hippocampal slices. Whereas these oscillations were reliably seen in interface slices, as previously reported (Fisahn *et al.*, 1998; Kubota *et al.*, 2003), no such activity persisted in submerged slices using standard flow rates (1.8–2.4 mL/min) either in the chamber standard or the modified submerged chamber (Figs 1A and B, and 2A). As the metabolic demand may be greater during ongoing network activity (Huchzermeyer *et al.*, 2008), we searched for submerged conditions with improved metabolic supply to allow the recording of network activities similar to those observed in interface chambers and the intact brain. We found that increasing the flow rate (5.2 mL/min) of the superfusion fluid in a recording chamber modified with an inert plastic insert to optimize laminar flow across the slice (Fig. 1A and B) allowed us to record both spontaneous sharp wave*–*ripples (Fig. 2Ai) and cholinergically induced fast oscillations (Fig. 2Aii), network activities that are known to be generated intrinsically within CA3 of the hippocampus *in vivo* (Buzsaki, 1986; Csicsvari *et al.*, 2003). These network activities were only rarely seen in CA1, even at high superfusion rates. CA1 sharp wave–ripples were seen in one out of five slices, whereas CA1 fast oscillations were seen in two out of nine slices. However, using the dual superfusion chamber, even with a flow rate of 3–3.5 mL/min for each channel (Fig. 1C–F), we readily observed in CA1 both spontaneous sharp wave*–*ripples propagated from the CA3 local network (six out of nine slices; Fig. 2Bi) and prominent cholinergically induced oscillations (six out of eight slices) (Fig. 2Bii).

### Superfusion rate correlates with local oxygen saturation

**Fig. 3:**
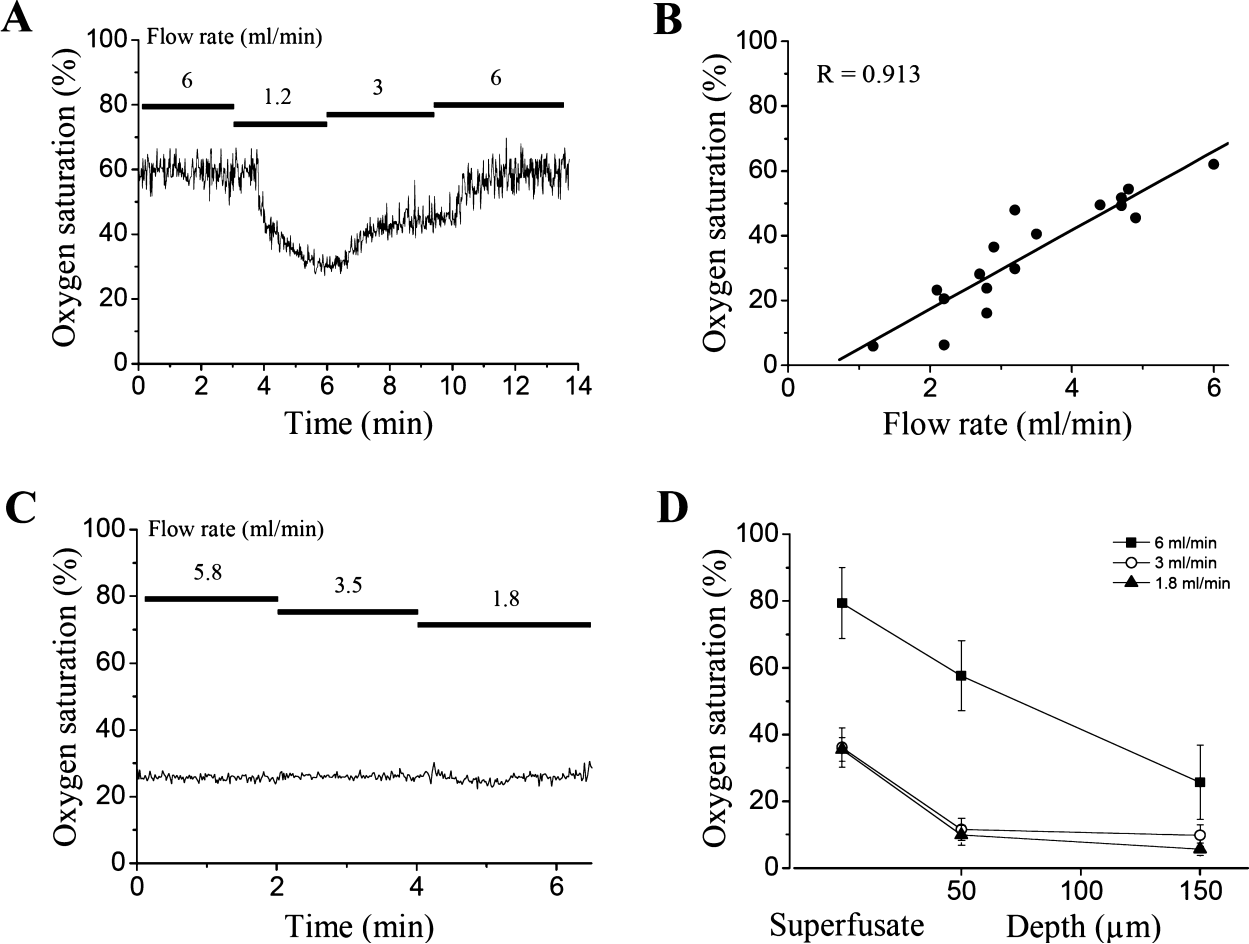
Relationship between flow rate and oxygen saturation in a modified submerged slice chamber. (A) The effect of flow rate on oxygen saturation measured 50–100 μm above CA3 of a submerged hippocampal slice. (B) Oxygen saturation as a function of flow rate measured at room temperature (*n*=5 slices; two to three data points per slice). Polyethylene tubing with substantial permeability for O_2_ was used in these experiments. (C) The oxygen measurement was not sensitive to changes in the flow rate of non-bubbled solution. (D) Oxygen saturation as measured using carbon-fibre voltammetry 50–100 μm above, and 50 μm and 150 μm below, the upper surface of the slice at flow rates of 6, 3 and 1.8 mL/min. Similarly to the optode measurements, a reduction in oxygen saturation was observed in the perfusate at 3 and 1.8 mL/min, respectively. At 50 μm depth within the tissue, at perfusion speeds of 6, 3 and 1.8 mL/min, oxygen saturation was reduced to 58 ± 10%, 12 ± 3% and 10 ± 3%, respectively. At 150 μm, this was reduced further to 26 ± 11%, 10 ± 3% and 6 ± 2%, respectively (mean ± SEM). Tygon tubing with low permeability for O_2_ was used in this experiment.

We hypothesized that an important difference between low and high flow rates could be variation in the oxygen saturation of the superfusion solution in the recording chamber, which would influence the metabolic state of the slice. To test this idea, we first measured the oxygen saturation in the ACSF immediately above the slice while the flow rate was altered (Fig. 3A). The oxygen saturation changed almost linearly with flow rate (*R*=0.913, *P*<0.01, Pearson’s correlation, *n*=5 slices; Fig. 3B). To exclude the possibility that the oxygen probe could be sensitive to changes in flow rate by itself, we superfused ACSF equilibrated with air using different flow rates. Changes in flow rate did not affect the oxygen measurement (Fig. 3C). As the oxygen saturation within the tissue differs from that of the superfusate (Foster *et al.*, 2005), we also measured the oxygen saturation above the slice and within the slice at depths of 50 μm and 150 μm using carbon-fibre voltammetry at flow rates of 6, 3 and 1.8 mL/min (*n*=5 slices; Fig. 3D). Measurement of oxygen level immediately above the tissue showed a reduction in oxygen saturation from 79 ± 11% at 6 mL/min to 36 ± 6% and 35 ± 4% at 3 mL/min and 1.8 mL/min, respectively. Oxygen saturation decreased steeply between 50 and 150 μm inside the tissue. At 150 μm depth within the slice, there were slightly hyperoxic conditions (26 ± 11%) at 6 mL/min, whereas hypoxic conditions (10 ± 3% and 6 ± 2%) were seen at 3 and 1.8 mL/min.

### Importance of oxygen supply for the maintenance of sharp wave–ripple oscillations

**Fig. 4:**
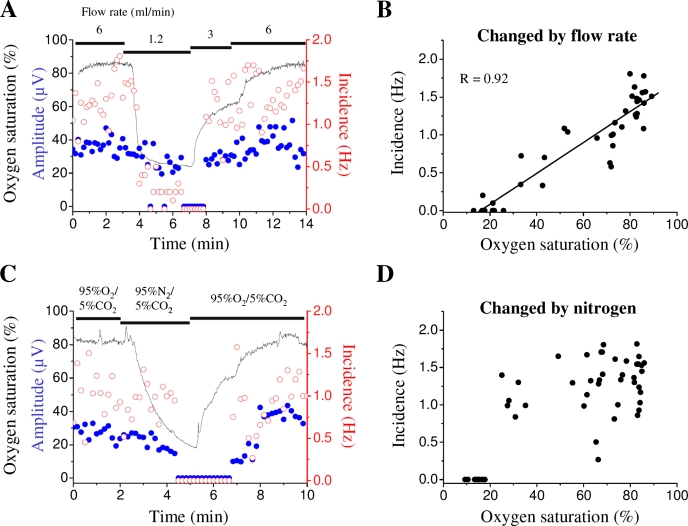
Importance of oxygen levels for maintenance of spontaneous sharp wave*–*ripples. (A) Effect of flow rate on oxygen saturation and incidence of sharp wave*–*ripples recorded with a planar 8 × 8 microelectrode array. Black trace, oxygen saturation (%) during experiment; open circles, incidence of sharp wave*–*ripples (Hz); filled circles, amplitude of sharp wave*–*ripples (μV). Note the reduction of the incidence of sharp wave*–*ripples with lower oxygen saturation. The incidences were 1.1 ± 0.1 Hz, 1.3 ± 0.1 Hz and 0.4 ± 0.14 Hz (mean ± SEM) at flow rates of 6, 3 and 1.2 mL/min, respectively. The incidence at 1.2 mL/min was significantly lower than that at either 6 or 3 mL/min (both *P*<0.01, paired Student’s *t*-test, *n*=4 slices). The corresponding amplitudes were 28.0 ± 7.4 μV, 27.0 ± 8.2 μV and 23.3 ± 6.3 μV, respectively (*P*>0.1, paired Student’s *t*-test, *n*=4 slices). (B) Plot of incidence of sharp wave*–*ripples against oxygen saturation modified by reduced flow rate (*n*=4 slices; five or six data points per slice). Least-squares line fit superimposed. (C) Superfusion of the slice with artificial cerbrospinal fluid bubbled with 95% N_2_/5% CO_2_ abolishes sharp wave*–*ripples. Black trace, oxygen saturation (%) during the experiment; open circles, incidence of sharp wave*–*ripples (Hz); filled circles, amplitude of sharp wave*–*ripples (μV). The incidences were 1.3 ± 0.1 and 0.3 ± 0.2 Hz (mean ± SEM; *P*<0.01, paired Student’s *t*-test, *n*=4 slices) with 95% O_2_/5% CO_2_ and 95% N_2_/5% CO_2_, respectively. The corresponding amplitudes were 17.5 ± 3.1 μV and 16.3 ± 2.4 μV (*P*>0.1, paired Student’s *t*-test, *n*=4 slices). (D) Plot of incidence of sharp wave*–*ripples against oxygen saturation modified by bubbling with 95% N_2_/5% CO_2_ (*n*=4 slices; five or six data points per slice). Tygon tubing with low permeability for O_2_ was used in these experiments.

To investigate the relationship between flow rate, oxygen saturation and sharp wave*–*ripple activity, we monitored both oxygen saturation and the incidence and amplitude of sharp waves while altering the flow rate (Fig. 4A). Reducing the superfusion rate from 6 to 1.2 mL/min caused a rapid reduction in the incidence of sharp wave–ripples, and there was a strong correlation between the incidence of sharp wave*–*ripples and the measured oxygen saturation (*R*=0.92, *P*<0.01, Pearson’s correlation, *n*=4 slices; Fig. 4A and B). To test whether oxygen saturation was a causal factor, we repeated the experiment at a constant high flow rate and altered the oxygen content of the superfusion solution by bubbling it with 95% N_2_/5% CO_2_. Again, we observed a rapid decrease in oxygen saturation of the ACSF, accompanied by a reduction in the incidence of sharp wave*–*ripples (*n*=4 slices; Fig. 4C and D). These data suggest that the spontaneous emergence of sharp wave*–*ripples in hippocampal slices critically depends on sufficient oxygen supply.

### Importance of oxygen supply for the maintenance of cholinergically induced fast network oscillations

**Fig. 5:**
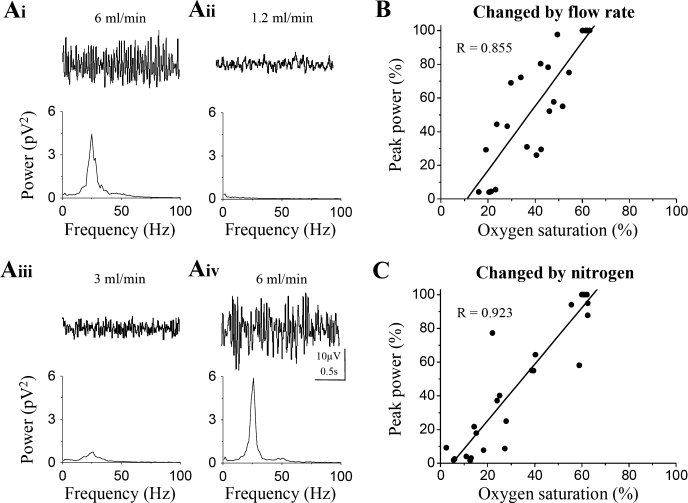
Importance of oxygen levels for the maintenance of cholinergically induced fast network oscillations. (Ai–iv) Temporal sequence of representative traces of rhythmic activity at different flow rates and their corresponding power spectra showing the sensitivity of network activity to oxygen levels. Oscillations were detected using a planar 8 × 8 microelectrode array. Peak power: 4.1 ± 1.8 pV^2^/Hz at 6 mL/min; and 0.02 ± 0.1 pV^2^/Hz at 1.2 mL/min (*P*<0.01, paired Student’s *t*-test, *n*=8). On return to a 6 mL/min flow rate, peak power returned to the control levels (4.8 ± 2.5 pV^2^/Hz; *P*>0.1, paired Student’s *t*-test, *n*=5). (B) Relationship between the oxygen saturation of artificial cerbrospinal fluid changed by flow rate and the power of the oscillation (*n*=8 slices; two or three data points per slice). Least-squares line fit superimposed. (C) Relationship between the power of oscillations and the oxygen saturation changed by bubbling of 95% N_2_/5% CO_2_ at a constant flow rate of 5.4 mL/min (*n*=8 slices; two or three data points per slice). Least-squares line fit superimposed. The power of the oscillations was normalized to the maximum value observed at 6 mL/min. Polyethylene tubing with substantial permeability for O_2_ was used in these experiments.

Next, we investigated whether the requirement of oxygen supply also holds for cholinergically induced fast network oscillations. Reducing the superfusion rate from 6 to 1.2 mL/min caused a rapid reduction in the power of fast oscillations, a change that could be reversed by increasing the flow rate (Fig. 5Ai–iv). Similarly, reducing the oxygen content of the superfusion solution by bubbling it with 95% N_2_/5% CO_2_ led to a rapid decrease in oxygen saturation of the ACSF and caused a parallel reduction in the power of fast network oscillations. There was a strong linear correlation between the power of network oscillations and the measured oxygen saturation in the chamber, both when modified by flow rate (*R*=0.855, *P*<0.01, Pearson’s correlation, *n*=8; Fig. 5B), and when modified by 95% N_2_/5% CO_2_ (*R*=0.923, *P*<0.01, Pearson’s correlation, *n*=8; Fig. 5C). These data show that not only the spontaneous emergence of network activity, but also pharmacologically induced oscillations, require sufficient oxygen supply.

### Importance of oxygen supply for the maintenance of GABAergic inhibition during cholinergically induced fast network oscillations

**Fig. 6:**
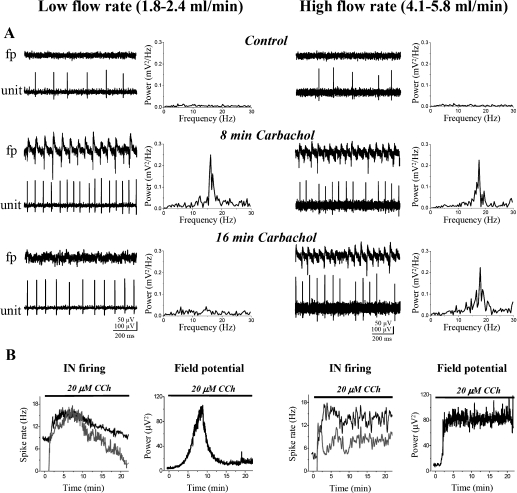
Dependence of the cholinergically enhanced interneuronal (IN) firing and network oscillation on the flow rate. (A) Concurrent recording of field potential (fp) with power spectrum and spiking activity of individual neurons in the stratum oriens (unit) of the hippocampal CA3 at different times after bath application of the cholinergic receptor agonist carbachol (CCh) (20 μm) at low (left) and high flow rates (right) in submerged rat slices. (B) Oscillatory power (10–22 Hz), spiking frequency (black, cells with spontaneous activity before application of carbachol; grey, neurons firing only in the presence of carbachol) plotted over time after the start of carbachol application during low (left) and high flow rate (right). Carbachol induced only transient oscillatory activity [peak frequency, 17.2 ± 0.4 Hz at room temperature; peak power, control, 3.9 ± 2.1 μV^2^/Hz (*n*=7); in drug (8 min), 98.1 ± 29.1 μV^2^/Hz (*P*<0.01, paired Student’s *t*-test); in drug (16 min), 17.6 ± 12.5 μV^2^/Hz (*P*>0.1, as compared to control using paired Student’s *t*-test)] in parallel with a transient increase of interneuronal firing during low flow rates. In contrast, both oscillations [peak frequency, 17.9 ± 0.6 Hz; peak power, control, 9.9 ± 4.1 μV^2^ (*n*=7); in drug (8 min), 83.5 ± 25.4 μV^2^ (*P*<0.01, paired Student’s *t*-test); in drug (16 min), 80.6 ± 19.9 μV^2^ (*P*<0.01, as compared to control using paired Student’s *t*-test)] and increased firing activity were persistent in the presence of carbachol at high flow rates.

Finally, in order to understand the underlying mechanisms of the oxygen demand, we investigated the effects of changing the flow rate on individual cells in the network. We have previously shown that carbachol-induced oscillations depend on GABAergic inhibition (Fisahn *et al.*, 1998; Pálhalmi *et al.*, 2004). We therefore investigated whether changes in flow rate also alter GABAergic inhibition. We recorded the spiking activity of visually identified inhibitory interneurons in the stratum oriens of CA3 (Fig. 6A). In control conditions, 46% (13/28) of tested cells fired action potentials at low flow rates (1.8–2.4 mL/min), whereas 66% (19/29) were active at high flow rates (4.1–5.8 mL/min). The average firing rate of the active neurons was 1.5 ± 1.0 Hz (*n*=13) and 2.3 ± 2.8 Hz (*n*=19) at low and high flow rates, respectively, which did not differ significantly (*P*>0.1, independent Student’s *t*-test).

At low flow rates, carbachol induced a transient increase in the firing rate of both spontaneously active (from 9.1 ± 3.2 Hz to 15.8 ± 3.9 Hz; *n*=3, *P*<0.01, paired Student’s *t*-test) and originally non-spiking (from 0 to 15.1 ± 4.3 Hz, *n*=4, *P*<0.01, paired Student’s *t*-test) neurons associated with fast oscillatory activity (Fig. 6B), but the oscillations disappeared rapidly and the firing rate decreased again within 20 min in the continued presence of carbachol (for spontaneously active cells, 10.2 ± 3.8 Hz, *P*>0.1, and for originally non-spiking neurons, 6.3 ± 4.1 Hz, *P*<0.05, as compared to control using paired Student’s *t*-test; Fig. 6B). In contrast, at high flow rates, an increased firing rate persisted for tens of minutes during superfusion of carbachol-containing solution in both spontaneously active neurons (from 4.2 ± 2.5 Hz to 12.9 ± 4.1 Hz, *n*=4, *P*<0.01, paired Student’s *t*-test; and remained after 16 min at 13.4 ± 3.5 Hz, *n*=4, *P*<0.01, as compared to control using paired Student’s *t*-test) and originally non-spiking neurons (from 0 to 6.1 ± 3.1 Hz, *P*<0.01, paired Student’s *t*-test; and remained after 16 min at 8.8 ± 4.3 Hz, *P*<0.01, as compared to control using paired Student’s *t*-test), associated with persistent fast network oscillations (Fig. 6A and B; see also Figure 1 in Hájos *et al.*, 2004). Thus, increasing the flow rate enhances the firing of interneurons and enables the maintenance of persistent synchronous network activities in slice preparations.

## Discussion

The main findings of the present study are as follows: (i) high laminar flow rate (4.1–6.0 mL/min) enables stable spontaneous and pharmacologically induced network activity in submerged hippocampal slices; (ii) this network activity is enabled by the increased oxygen supply provided by a higher flow rate in the modified submerged chamber; and (iii) interneurons can maintain their firing rate during cholinergic activation only with the higher flow rate.

The high sensitivity of hippocampal network activity to oxygen tension is consistent with other recent studies (Wu *et al.*, 2005; Huchzermeyer *et al.*, 2008). Earlier investigations into the cellular mechanisms underlying network oscillations have been hampered by the transient nature of the induced activity in submerged conditions (McMahon *et al.*, 1998; Kawaguchi, 2001; Gloveli *et al.*, 2005) as compared to interface conditions (e.g. Whittington *et al.*, 1995; Fisahn *et al.*, 1998; Sanchez-Vives & McCormick, 2000; Kubota *et al.*, 2003). We suggest that higher superfusion flow rate could ameliorate this problem.

In addition to the flow rate, the oxygen supply to the slice under submerged conditions will depend on several other factors, including the type of tubing used and the configuration of the slice chamber. Short tubing with minimal oxygen permeability (e.g. Tygon or Teflon) helps to maintain high oxygen levels (note the difference in the initial values of oxygen saturation using polyethylene and Tygon tubing in Figs 3A and 4A, respectively). We could reduce the flow rate and still observe oscillations by reducing the volume of the chamber and help to create laminar flow by using a custom-made inert plastic insert (Fig. 1A and B). Propagation of network oscillations from CA3 to CA1 was supported by superfusing the slice from both surfaces (Fig. 1C–F). Thus, the flow rate needed to ensure sufficient oxygen supply at a given recording condition could vary with several other technical factors.

The recognition that oxygen levels might be insufficient to support some energy-consuming neuronal functions in submerged slices at low flow rates, such as interneuronal firing activity during network oscillations, raises the question of whether baseline neuronal properties might also be significantly influenced by low flow rates. Reassuringly, a recent study showed that evoked local field potential responses could remain unaltered despite significantly reduced power of cholinergically induced gamma oscillations with reduced oxygen levels (Huchzermeyer *et al.*, 2008).

Although our results suggest that a relatively high oxygen level in the superfusion solution is necessary to maintain network activity in slices, one should be aware that hyperoxygenation of tissue could alter several parameters of neuronal operation and even induce acute cell death (Mulkey *et al.*, 2001; Pomper *et al.*, 2001). Thus, a number of studies have shown that the use of carbogen gas can increase oxygen tension within a slice to levels substantially higher (200–500 Torr) than have been measured in the intact brain (10–34 Torr) (Jiang *et al.*, 1991; Mulkey *et al.*, 2001). In our experiments, at 6 mL/min flow rate, oxygen tension was slightly elevated at 150 μm depth relative to air. One reason for the necessity of a higher oxygen tension *in vitro* might be the longer diffusion distances in the absence of blood circulation. Differences between slices and the intact brain in oxygen availability during neuronal activity have been discussed in detail in a recent review (Turner *et al.*, 2007).

## Conclusion

We conclude that naturalistic network activity can be studied under submerged conditions when a sufficient oxygen supply is maintained by a high flow rate of superfusion fluid. The exact flow rate required depends on several technical factors that are unique to each experimental setup. We suggest that measurement of oxygen could be a useful tool with which to optimize the experimental conditions, although several other factors might contribute, including *P*co_2_ and pH (e.g. Stenkamp *et al.*, 2001).

## Abbreviations

ACSF: artificial cerebrospinal fluid
